# Imaging of ectopic intrathoracic multinodular goiter with pathologic correlation: a case report

**DOI:** 10.4076/1757-1626-2-8554

**Published:** 2009-07-16

**Authors:** Mayia Pilavaki, George Kostopoulos, Anthoula Asimaki, Athanasia Papachristodoulou, Stilliani Papaemanouil, Panagiotis Palladas

**Affiliations:** 1 Department of Radiology, General Hospital “G.Papanikolaou”ThessalonikiGreece; 2 Department of Thoracic and Cardiovascular Surgery, General Hospital “G.Papanikolaou”ThessalonikiGreece; 3 Laboratory of Pathology, General Hospital “G.Papanikolaou”ThessalonikiGreece

## Abstract

**Introduction:**

We present a case of ectopic intrathoracic multinodular goiter and correlate the magnetic resonance imaging appearance with the histological findings.

**Case presentation:**

A 72-year-old man was referred to our institute with a two month history of cough. The chest radiograph showed a mass located in the mediastinum. A chest computed tomography scan, showed an enhancing mass at the right side of the middle mediastinum where magnetic resonance images, demonstrated a multicystic mass. The mass was excised through a right lateral thoracotomy and histologically it proved to be an ectopic multinodular goiter.

**Conclusions:**

Although ectopic intrathoracic multinodular goiter is a rare entity, it should be considered in the differential diagnosis of mediastinal masses. The preoperative diagnosis is important as, unlike substernal goiter which is surgically approached through the neck, the ectopic thyroid is treated by thoracotomy.

## Introduction

Ectopic thyroid tissue primarily occurs along the course of the embryologic migration of the thyroid gland. It has been found from the tongue to the diaphragm. Mediastinal thyroid may be differentiated into primary and secondary form. Primary mediastinal goiters, such as the case presented here, are quite rare, occurring in less than 1% of all goiters. The ectopic tissue derives its blood supply from intrathoracic vessels and thus, thoracotomy or sternotomy is required for resection of the mass. In contrast, the more common, secondary form represents large cervical goiters, which extent into the superior mediastinum and derives its blood supply from thyroid vessels in the neck [1]. For preoperative evaluation, chest radiograph, computed tomography (CT) and magnetic resonance (MR) images are effective for differential diagnosis of other mediastinal tumors.

## Case presentation

**Figure 1.**
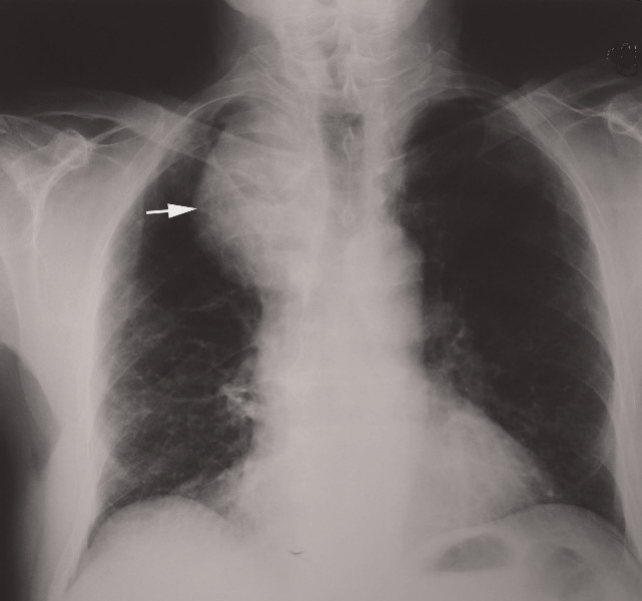
Posteroanterior chest radiograph shows a mass (arrows) in the mediastinum with deviation of the trachea.

**Figure 2.**
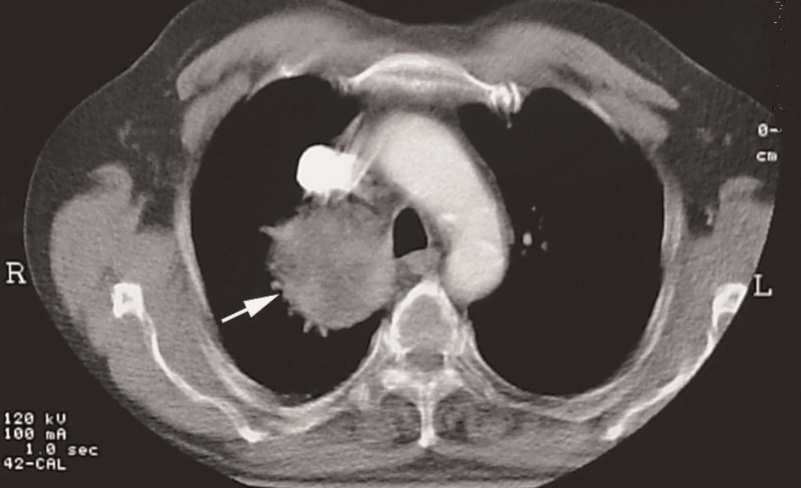
Contrast-enhanced CT demonstrates an inhomogeneous enhancing mass at the right side of the mediastinum (arrow).

**Figure 3.**
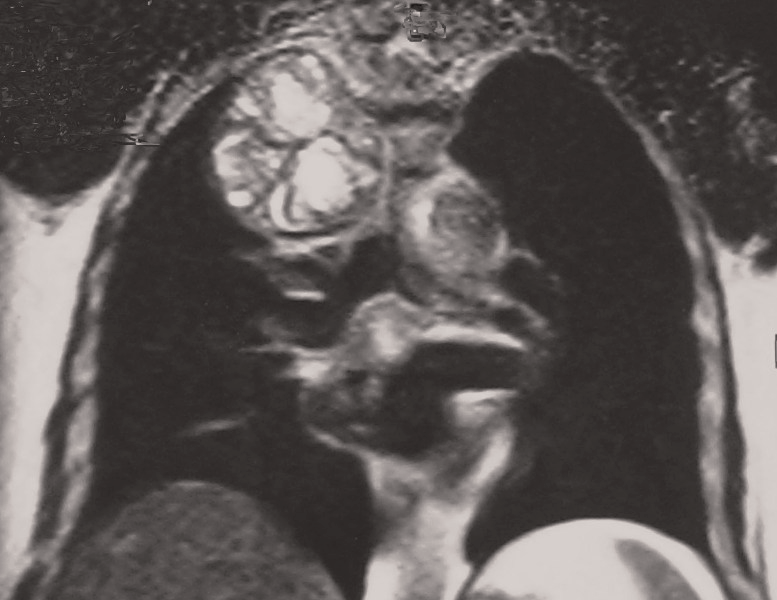
Coronal dark blood HASTE MR Image shows a multicystic mass with septa, in a cauliflower-like arrangement.

**Figure 4.**
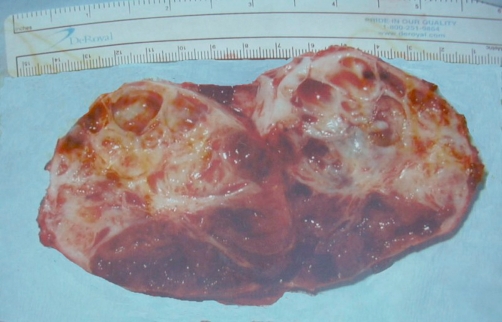
Photograph of the sectioned gross specimen shows cystic areas.

**Figure 5.**
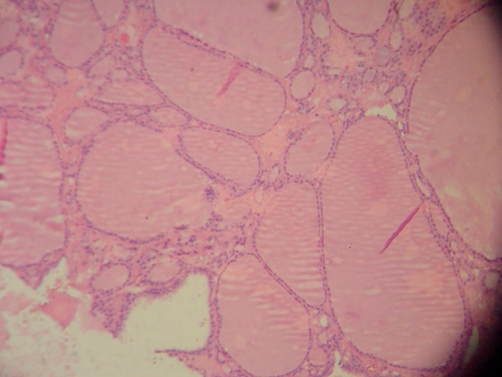
Microscopic view of the ectopic thyroid shows thyroid follicles containing colloid (Hematoxylin & Eosin stain, original magnification ×400).

A 72-year-old Greek man, smoker, presented with a two month history of cough. The physical examination was normal. The neck was supple without thyromegaly. Laboratory testing showed no abnormalities and thyroid function tests were normal. The chest radiograph showed a sharply demarcated mass located in the mediastinum with deviation of the trachea (Figure 1). CT, after the administration of contrast medium, showed an enhancing mass at the right side of the middle mediastinum, behind the great vessels, extending to the level of the bifurcation of the trachea (Figure 2). MR images, demonstrated a multicystic mass consisting of hyperintense areas scattered throughout a background of intermediate signal intensity, rendering the lesion a cauliflower-like appearance, especially on T2-weighted images (Figure 3). Neither CT nor MR identified a pedicle connecting the mass to the cervical region. The mass was excised through a right lateral thoracotomy. Intraoperative findings revealed that there was no glandular continuity between the thyroid gland and the mass and that the blood supply of the tumor derived from intrathoracic vessels. Gross examination of the specimen showed an encapsulated global mass, measuring 7.5 cm in diameter (Figure 4). On cut sections the mass appeared cystic with elastic consistency. On histologic section it was composed of colloid distended follicles intermixed with foci of hyperplasia and separated by dense hyalinizing bands of fibrous tissue (Figure 5). The histopathological findings were compatible with a colloid multinodular goiter.

The postoperative course of the patient was uncomplicated. Twelve months after the surgery he is asymptomatic and disease free.

## Discussion

The thyroid tissue starts developing during the fourth embryonic week. It appears on the tongue as an epithelial growth. By the seventh embryonic week, the thyroid gland descends to the adult position, anterior to the trachea. Ectopic thyroid gland can be found along this pathway, as in the region of the neck, the mediastinum, the pharynx, the larynx, the esophagus, the trachea, and around the aorta [2-4]. Lingual thyroid is the most common location of ectopic thyroid tissue, accounting for 90% of the cases [3].

Ectopic intrathoracic thyroid is a rare presentation of thyroid disease, and only a few cases have been reported in the literature [5-7]. It is believed that mediastinal thyroid tissue represents accessory ectopic tissue from the median thyroid anlage. During embryogenesis, as the heart descents, the thyroid is drawn caudally, and such ectopic mediastinal thyroid tissue is thought to be caused by abnormal mechanical relationships to the heart. Thereby, primary thoracic masses represent portions of the gland that remained attached to the pericardium or great vessels [1].

Patients with ectopic intrathoracic goiter are usually asymptomatic with the tumor reported as incidental finding on chest radiograph [3]. Sometimes they may present with respiratory symptoms like our patient.

Findings on chest radiography include a soft tissue mass, calcification and tracheal displacement [3]. Chest CT provides important information about the location of the mass and its relation with the great vessels [5]. In the case of our patient the diagnosis of the ectopic intrathoracic multinodular goiter was based on the MR findings. The multicystic, cauliflower-like arrangement on MR examination reflected the histological pattern of the lesion as it was composed of colloid distended follicles intermixed with foci of hyperplasia and separated by dense hyalinizing bands of fibrous tissue. The shape and the size of the follicles vary widely.

The differential diagnosis of middle mediastinal masses includes lymphadenopathy, primary tracheal or esophageal tumors, bronchogenic cysts and esophageal duplication cysts.

Ectopic intrathoracic thyroid should be removed to rule out malignancy [5]. Thoracotomy or sternotomy is usually required.

## Conclusions

This case establishes that ectopic intrathoracic multinodular goiter should be considered in the differential diagnosis of mediastinal masses. The preoperative diagnosis is essential and is based on MR images findings. Thoracotomy or sternotomy is required for resection of the mass and prognosis is excellent following a successful excision.

## Abbreviations

CT: Computed tomography
MR: Magnetic resonance
